# Supplementary material for: A parabrachial to hypothalamic pathway mediates defensive behavior
Source: eLife. 2023 Mar 17;12:e85450. doi: 10.7554/eLife.85450 (PMC10023160; doi:10.7554/eLife.85450)
Supplement: Figure 6—source data 2. [file elife-85450-fig6-data2.docx]

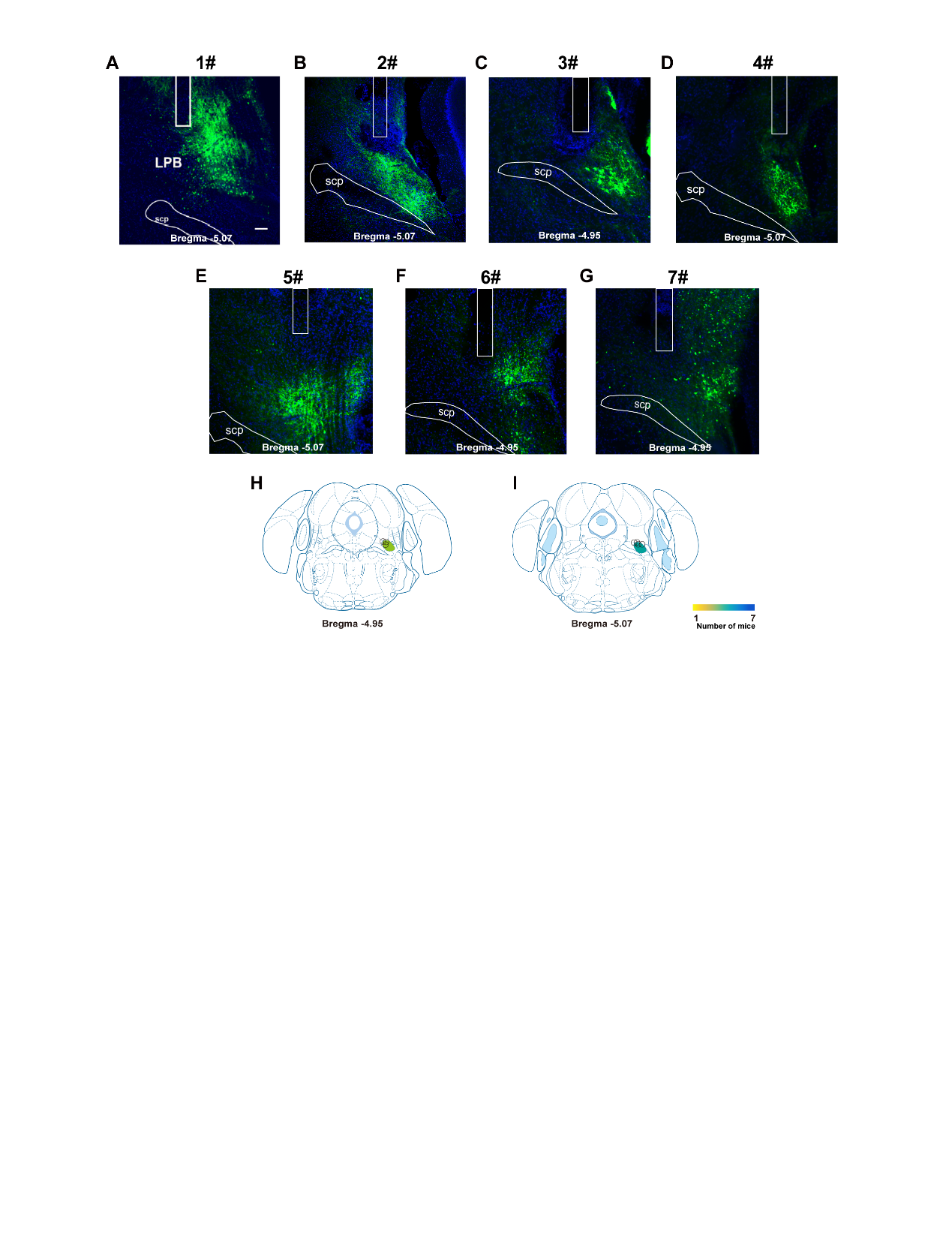


**Figure 2.** Images of ChR2-EYFP expression in the LPB and optical fiber implantation above the LPB (A-G), with circles (H-I) indicating the location of optical fibers.


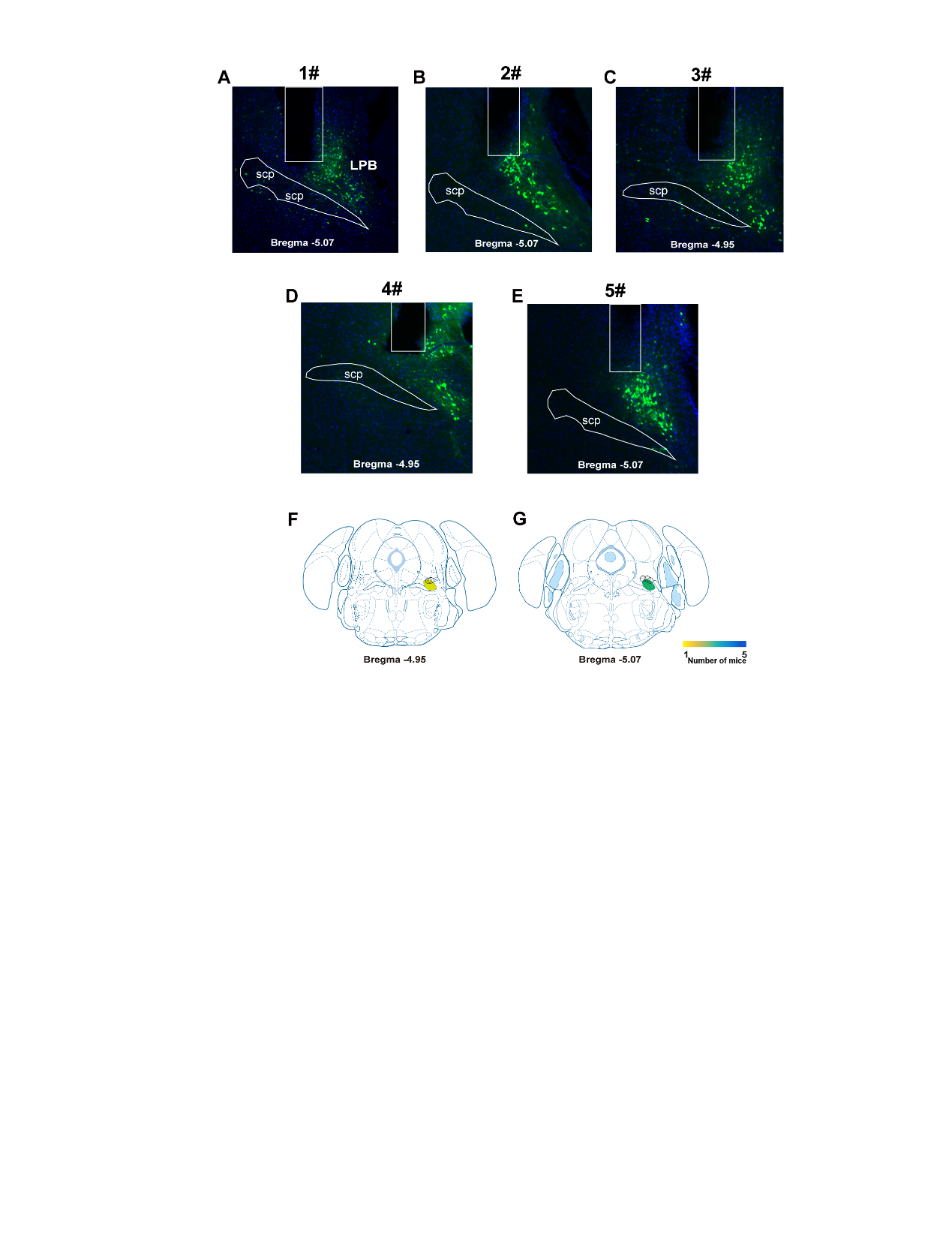


**Figure 3.** Images of Gcamp7s expression in the LPB and optical fiber implantation in the LPB (A-E), with circles (F-G) indicating the location of optical fibers.


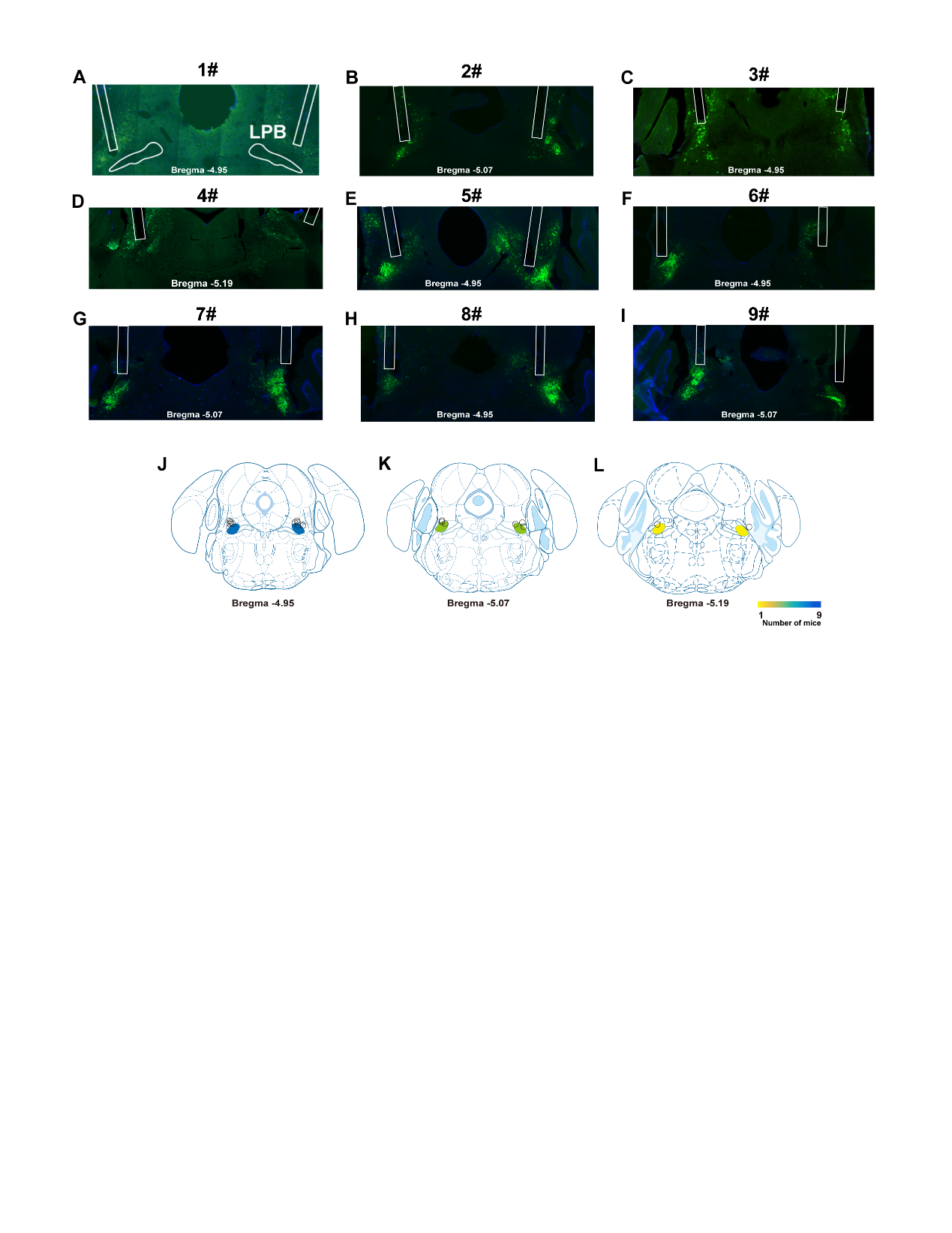


**Figure 4.** Images of GtACR1-EYFP expression in the LPB and optical fiber implantation above the LPB (A-I), with circles (J-L) indicating the location of optical fibers.


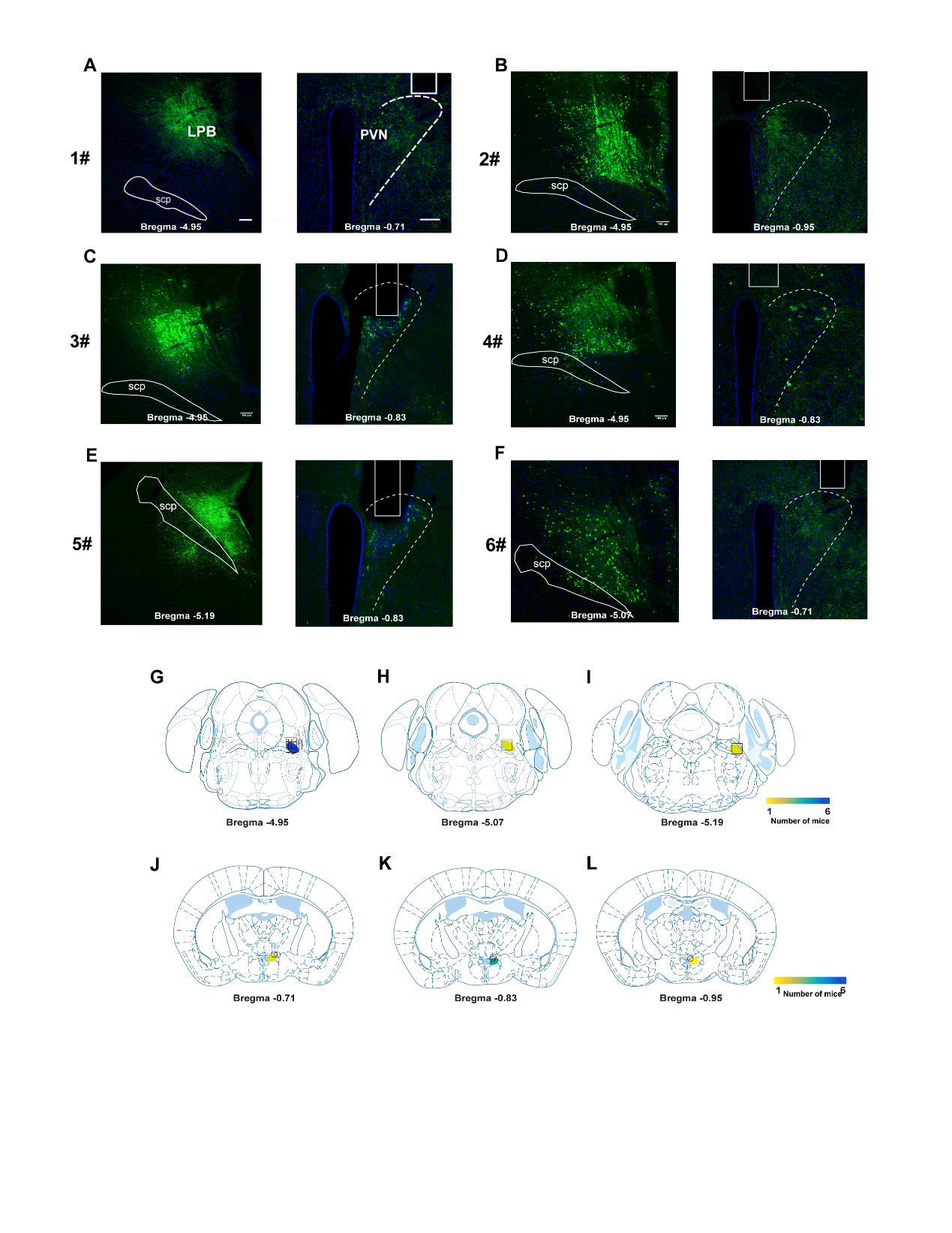


**Figure 5.** Images of ChR2-EYFP expression in the LPB and optical fiber implantation above the PVN (A-F). The square boxes indicate the location of virus expression and circles represent optical fiber location (G-L).


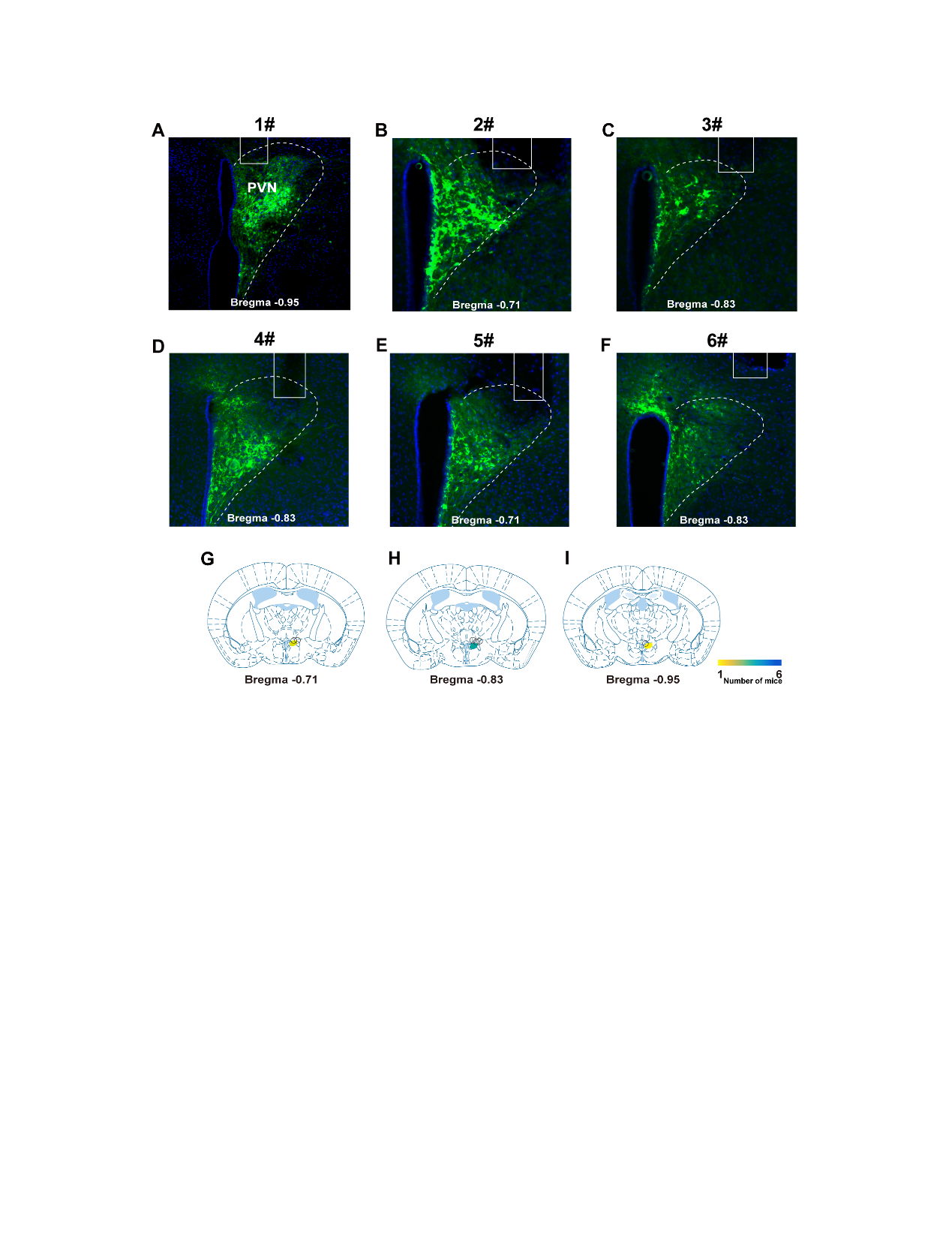


**Figure 6.** Images of ChR2-EYFP expression in the PVN and optical fiber implantation above the PVN (A-F). The circles represent optical fiber location (G-I).
